# The advent of a pan-collagenous CLOVIS POINT for pathotropic targeting and cancer gene therapy, a retrospective

**DOI:** 10.3389/fmmed.2023.1125928

**Published:** 2023-02-28

**Authors:** Erlinda M. Gordon, Frederick L. Hall

**Affiliations:** ^1^ Counterpoint Biomedica LLC, Santa Monica, CA, United States; ^2^ Delta Next-Gene, LLC, Santa Monica, CA, United States

**Keywords:** pan-collagen-binding domain, wound healing, stem cell capture, cancer gene therapy, immunotherapy, deltarex-G, CCNG1

## Abstract

The ‘Clovis Point’—an enabling prehistoric gain-of-function in stone-age tool technologies which empowered the Paleoindian-Americans to hunt, to strike-deep, and to kill designated target megafauna more efficiently—was created biochemically by molecular-genetic bio-engineering. This Biomedical “Clovis Point” was crafted by adapting a broad-spectrum Pan-Collagen Binding Domain (Pan-Coll/CBD) found within the immature pre-pro-peptide segment of Von Willebrand Factor into a constructive series of advanced medical applications. Developed experimentally, preclinically, and clinically into a cutting-edge Biotechnology Platform, the Clovis Point is suitable for 1) solid-state binding of growth factors on collagenous scaffolds for improved orthopedic wound healing, 2) promoting regeneration of injured/diseased tissues; and 3) autologous stem cell capture, expansion, and gene-based therapies. Subsequent adaptations of the high-affinity Pan-Coll/CBD (exposed-collagen-seeking/surveillance function) for intravenous administration in humans, enabled the physiological delivery, aka Pathotropic Targeting to diseased tissues *via* the modified envelopes of gene vectors; enabling 4) precision tumor-targeting for cancer gene therapy and 5) adoptive/localized immunotherapies, demonstrating improved long-term survival value—thus pioneering a proximal and accessible cell cycle control point for cancer management—empowering modern medical oncologists to address persistent problems of chemotherapy resistance, recurrence, and occult progression of metastatic disease. Recent engineering adaptations have advanced the clinical utility to include the targeted delivery of small molecule APIs: including taxanes, mAbs, and RNA-based therapeutics.

## 1 Introduction

With the advantages of retrospection, we are privileged to reflect on significant clinical progress and to honor our many colleagues and collaborators who participated in this enduring epic of gene discovery and biochemical pathway characterization which connects the conceptual principles of stem cell biology, signal transduction, protein phosphorylation, wound-healing, inflammation, and co-carcinogenesis to the driving oncogene addictions and the key rate-limiting growth factors and tumor suppressors governing mammalian cell cycle control. Herein, in the context of this review, we present the allegory of the ‘*Clovis Point*’—a prehistoric gain-of-function in stone-age tool making technologies which empowered the Paleoindian-Americans to hunt, to strike-deep, and to kill designated target megafauna more efficiently—as an instructive allegory and an enabling biotechnology platform. Another useful perspective and unifying concept is a selective focus on the *‘Proline-Directed Protein Kinases’* (Liu and Kipreos, 2000; Kannan and Neuwald, 2004) —40 serine/threonine kinases demonstrating a preference for proline at the P +1 position (Vulliet et al., 1989; Hall et al., 1990; Zhu et al., 2005) — that is, a selective, sharp focus on the family of mitogen-activated protein kinases (MAPKs) which mediate receptor-signaling events (Willams et al., 1993; Gordon et al., 1996; Hall et al., 1996); and the cyclin-dependent protein kinases (CDKs), targeted (as heterodimers) by the cognate “cyclin partner” to specific substrate proteins (Hall et al., 1991; Elledge et al., 1992; Peeper et al., 1993), and which mediate a myriad of intricate protein-protein interactions (PPIs) in biochemical pathways that regulate stem cell growth, proliferative competence, the cell division cycle, sustained survival, and differentiation processes under physiological conditions. The characterization of mitogenic signal transduction, from growth factor receptors to the immediate early events of gene expression, provides mechanistic understanding and new molecular tools for both regenerative and interventional medicine.

## 2 Solid-state binding of bioactive growth (and survival) factors on collagenous scaffolds

**FIGURE 1:**
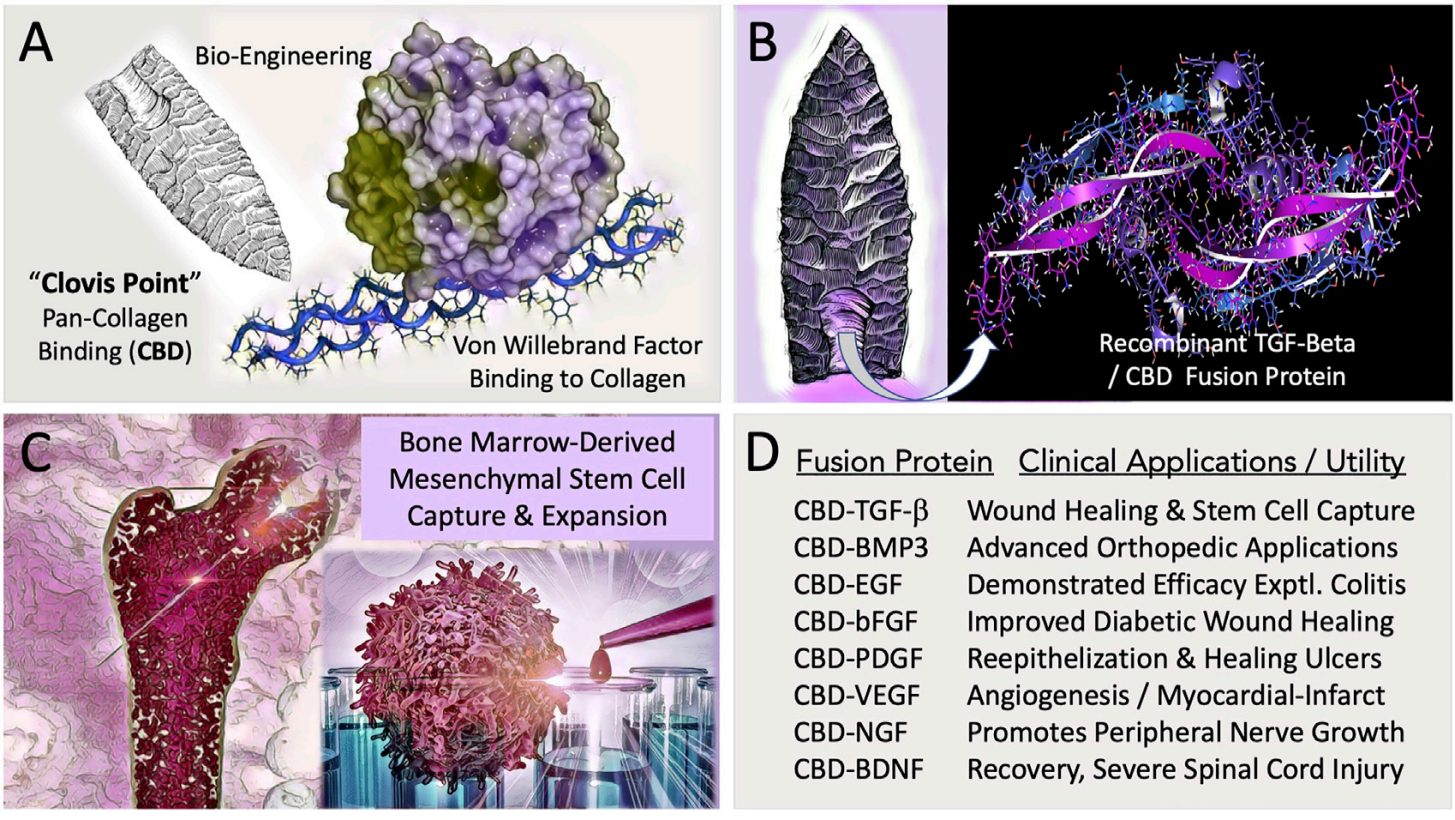
Engineering and Potential Clinical Utility of Collagen Binding (CBD) Growth Factors. **(A)** Concept of “hafting” a Pan-Collagen Binding Domain (CBD)—a molecular “Clovis Point”—by adaptive bio-engineering of vWF. **(B)** Chimeric growth factor protein expression in microorganisms, followed by purification and renaturation of the recombinant fusion polypeptides, (e.g., CBD-BMP3) provided the abundant bioactive materials needed for preclinical studies and translational research. **(C)** Graphic figure depicts the capture, expansion, conditioning, and differentiation of bone-marrow derived pre-hematopoietic, pre-mesenchymal stem cells for cell- and gene-based therapies. **(D)** Examples of collagen-targeted growth factors, highlighting advances in the potential utility of the biotechnology platform in the form of preclinical demonstrations.

To facilitate orthopedic wound healing and to promote the regeneration of injured tissues, a Biomedical “Clovis Point” was crafted by adapting a broad-spectrum Pan-Collagen-type Binding Domain (Pan-Coll/CBD) found within the immature pre-pro-peptide segment of Von Willebrand Factor into a constructive series for advanced medical applications (Figure 1). The conceptual bioengineering, expression, purification, and refolding/renaturation of the first collagen-binding TGF-β (and related) fusion proteins from microorganisms, enabled high-yield, cost-effective bioproduction of recombinant human TGF-β/BMP fusion proteins for preclinical studies: wherein enhanced osteogenesis was observed, as was the migration, growth, and differentiation of bone marrow mesenchymal cells (Tuan et al., 1996; Han et al., 1997; Andrades et al., 1999; Han et al., 2002). Preclinical studies included a collagen-targeted fibroblast growth factor (CBD-bFGF) which was shown to be effective in a diabetic wound healing model (Andrades et al., 2000; Andrades et al., 2001); and epidermal growth factor (CBD-EGF) which was shown to be effective in an animal model of experimental colitis (Hall et al., 2000a). The histological observations of exceedingly primitive stem cells migrating into growth-factor functionalized (yet acellular) collagen fiber scaffolds (Andrades et al., 1996), prompted a series of experiments on human bone marrow, where the clinical utility of CBD-TGF-β is acting as a “survival factor,” was used for the selective capture and characterization of a pre-hematopoietic, premesenchymal stem cell (Hall et al., 2001), presented clinically as an autologous stem cell platform for retrovirus-mediated gene therapy, exemplified by cell-based delivery and engraftment of the missing coagulation factor(s) of hemophilia (Gordon et al., 1997).

We celebrate the advancement of the conceptual platform in its ongoing translation to the clinic, along with the innovative variety of chimeric fusion proteins, collagen binding domains, and related binding motifs, as well as the increasing arrays of functionalized matrices, bandages, sponges, hydrogels, and membranes that have since been developed and proven in principle (Addi et al., 2017). Most notably, in cardiovascular disease: where a myocardial patch made of collagen membranes loaded with collagen-binding human vascular endothelial growth factor (CBD-VEGF) was shown to accelerate healing of the injured rabbit heart (Gao et al., 2011); and new approaches now include the selection and preconditioning of autologous mesenchymal stem cells with defined growth factors to improve the homing ability, engraftment, and survival of both the implanted cells and the ischemic cardiac tissue (Matta et al., 2022). Encouraging progress has been made in the translation to neurology and spinal cord injury: where collagen-based scaffolds and mesenchymal stem cells are employed with success in the experimental treatment of spinal cord injuries in humans and in animal models (Zachariou et al., 2022). Indeed, in traumatic models involving complete spinal cord transection the application of a linear collagen scaffold loaded with a collagen-binding fusion construct of a brain-derived neurotropic factor (CBD-BDNF) i) enhances functional recovery of a severed spinal cord by facilitating peripheral nerve infiltration and ingrowth (Han et al., 2014) and ii) enables neural stem cell capture and growth-factor conditioning for severe spinal cord injury repair (Li et al., 2016).

## 3 Going mobile: The pathotropic targeting of gene therapy vectors to diseased tissues

At the turn of the century, the promise of tumor-targeted gene therapies was generally considered a highly desirable yet elusive clinical goal—(Researchers Get a Dose of Reality as Logistics Stymie Gene Therapy (Langreth and Moore, WSJ, 27 Oct 1999). Indeed, the large number and diversity of collagen proteins, which includes some 28 family members (Ricard-Blum, 2011), make physiological targeting of a specific type of exposed collagen protein problematic. This problem is made even more complex with the realization that both cardiovascular injury and the stroma of invasive metastatic cancers are associated with high expression of extracellular signaling molecules, including collagen-triple-helix- containing-protein 1 (Mei et al., 2020): which activates an array of extracellular matrix metalloproteases that degrade and thereby alter the collagenous proteins (a proteolytic anaplasia), modifying the extracellular matrix of the vascular lesion, and creating what amounts to a collagenous anaplastic “jailbreak” of sorts for transformed cancer stem cells undergoing epithelial to mesenchymal transition (EMT). Fortunately, a unique and clinically useful feature of the high-affinity Pan-Collagen-type CBD-Growth Factor constructs and chimeric fusion proteins we originally developed for collagenous bandages, stem cell capture, and wound healing applications, was a demonstrable and advantageous pan-collagenous protein binding property (including denatured gelatin), which was adaptively engineered by experimentation into a high-affinity lesion-targeting property or gain-of function transposed into the protein envelopes of various experimental gene therapy vectors (Hall et al., 2000b; Gordon et al., 2002)— demonstrating the remarkable lesion-targeting gain-of-function in preclinical studies of vascular restenosis following balloon injury (Xu et al., 2001), and in animal models of metastatic cancer (Gordon et al., 2000).

## 4 A proper genetic payload: Proto-oncogene discovery and biochemical pathway characterization

**FIGURE 2:**
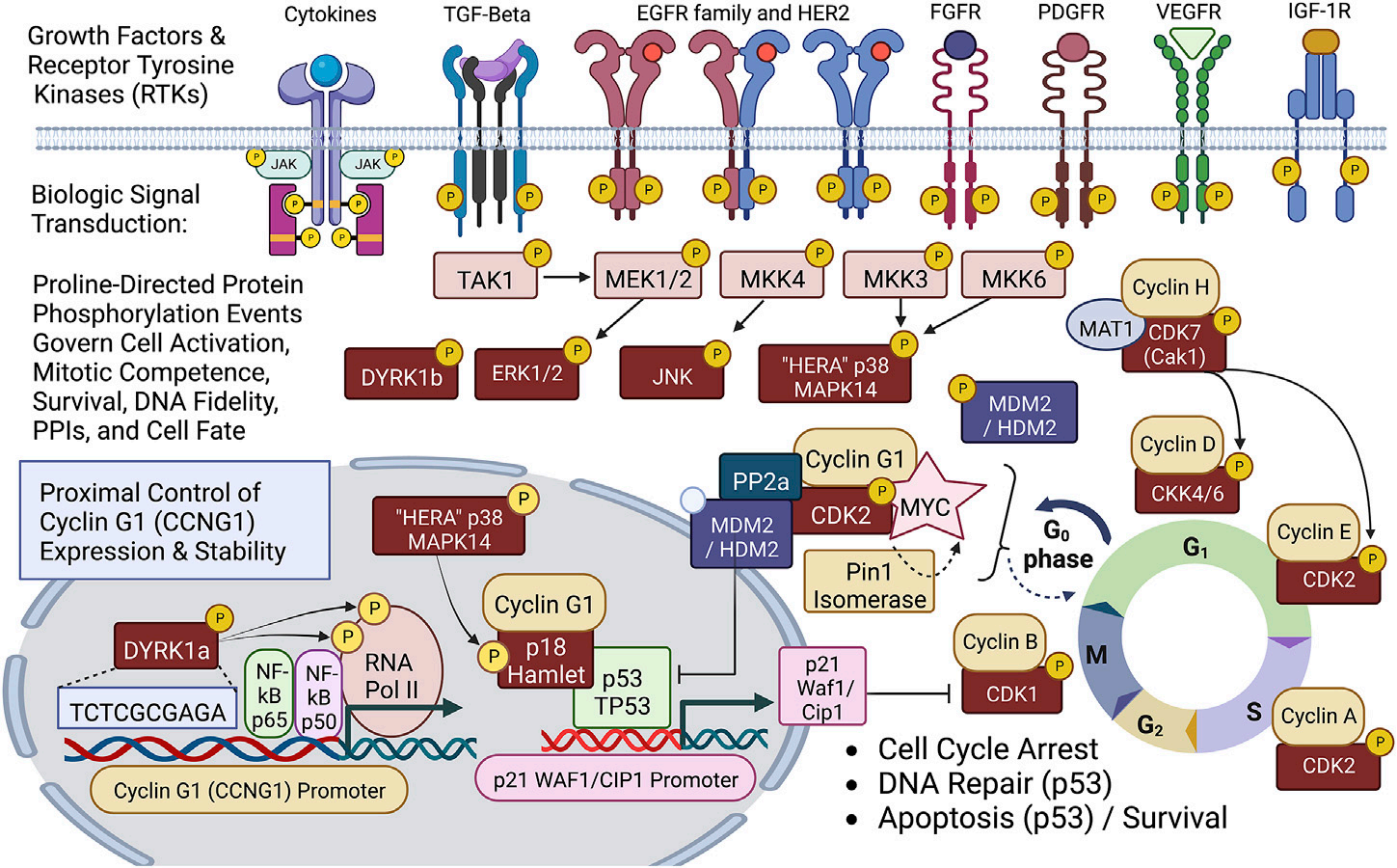
Diagram of site-specific proline-directed protein phosphorylation events governing stem cell activation: Identifying the proximal bio-cybernetics, driving oncogenes, and executive enzymology at the foundations of stem cell activation, cellular transformation, stem cell survival, sustained proliferation, and cell cycle control. The diagram highlights the executive regulatory flow of site-specific protein phosphorylation events linking the activation of major growth factor receptors to a series of mitogenic proline-directed signal transductions cascading through the “Hera kinase” (p38, human EGF-receptor- associated Mapk14) and DYRK1a, directing immediate early events leading to the activation of the Cyclin G1 (CCNG1) promoter and expression of the Pivotal Cyclin G1/Cdk2/Myc/Pin1/Mdm2/HDM2/p53 Axis of cell cycle control. Note: Cyclin G1 interacts physically with p18 Hamlet, a transcriptional activator and target substrate of p38 Hera/Mapk14, which mediates p53-dependent transcriptional responses to DNA damage and genotoxic stress in proliferative cells. Moreover, p38 Mapk14 and p18 Hamlet are implicated in chromatin-remodeling and transcriptional activation complexes associated with muscle differentiation. See text for additional details.

A simplified schematic view of biological signal transduction: from growth factor receptors, through receptor mediated tyrosine phosphorylation(s) and characteristic phospholipid effects, is shown as Figure 2, identifying the levels of organization and executive protein kinases involved. Our collaborative contributions to the field of biocybernetics, specifically proline-directed protein phosphorylation, include the identification and characterization of the Hera kinase, as a consort of the human EGF-receptor (Williams et al., 1993). This human-EGF-Receptor-associated protein kinase, designated p38α MAPK14, plays a major role regulating EGF-receptor function(s), stress responses, tumor dormancy, chemo-resistance, and cancer stem cell (CSC) survival (Martínez-Limón et al., 2020; Kudaravalli et al., 2022). Biochemical characterization and cloning of the human cyclin-dependent kinase activating kinase CAK1 (Yee et al., 1995; Yee et al., 1996), and its obligatory assembly factor, ménage à trois, MAT1 (Wang et al., 2000), provided additional mechanistic links to DNA replication and transcription, as well as the executive enzymology functioning upstream of the Cyclin/Cdk complexes governing cell cycle control.

The molecular cloning of the first human cyclin G-type gene (Wu et al., 1994), followed by studies of its overexpression and survival function in osteosarcoma cells—which lack a functional/wild type p53 (TP53) tumor suppressor (Wu et al., 1995)—provided a conceptual missing link in the proximal/initiating/transcriptional pathways driving the immediate early molecular genetics of neoplastic transformation and oncogenesis. While Cyclin G1 is a transcriptional target of p53 (TP53), the major tumor suppressor, the CCNG1 gene is overexpressed in many cancer cells in the absence of a functional p53, which led to the hypothesis and eventually the experimental confirmation that Cyclin G1 (CCNG1) represents a valid, proximal, and transforming proto-oncogene in its own right. Thus, the Cyclin G1 oncoprotein represents a singular strategic target and a *proximal point* of therapeutic intervention in the clinical management of human cancers. While antisense constructs of Cyclin G1, antisense Cyclin D1, and enforced expression of p21, were each found to be effective as transgenes (payloads) in blocking cancer cell survival *in vitro*, Cyclin G1 was selected for clinical development in a series of truncation experiments, using both point mutations and deletion mutants, to discern a potent dominant-negative “blocking” construct of the Cyclin G1 pathway, which was determined to be essential for the survival and proliferation of cancer cells derived from each of the three germ layers. Thus, a retroviral expression vector bearing an inhibitory construct of the Cyclin G1 (CCNG1) gene was developed (DeltaRex-G) and deployed for clinical gene therapy trials, where broad-spectrum bioactivity was observed (Gordon and Hall, 2010).

## 5 The pathotropic targeting platform enables gene therapy/immunotherapy

In terms of pathotropic tumor targeting, the pan-collagen binding *Clovis point* represents a powerful and enabling biotechnology for therapeutic gene delivery by its active and selective partitioning to injured and/or diseased tissues, including advanced metastatic cancers *via* exposed collagen. In terms of validating the strategic therapeutic ‘*payload*,’ it was, indeed, the demonstrated anti-cancer activities of DeltaRex-G (i.e., dnG1 enforced expression) observed *in vivo*, in advanced cases of chemo-resistant metastatic cancers, wherein the first blessings of remission were observed, following repeated DeltaRex-G infusions, and the first demonstrations of improved long-term survival were achieved. Moreover, the versatility of the gene delivery platform (simple intravenous infusions) enabled the first clinical demonstrations of localized (tumor-targeted), i.e., personalized cancer vaccinations *in situ*—using granulocyte macrophage colony stimulating factor (GMCSF) as a first immune-stimulating payload (pulsed, following DeltaRex-G tumor debulking)—where again, significant improvements in the survival of end-stage patients were demonstrated (Gordon et al., 2008; Ignacio et al., 2010). Detailed histological examinations of immune cell trafficking within the tumor microenvironment of DeltaRex-G-treated tumors revealed the selective killing (apoptosis) of cancer cells, tumor-associated stromal elements, and the proliferative cells of tumor neo-vasculature, while preserving of a patient’s innate tumor surveillance function (Stendhal-Dy et al., 2018), which appears favorable for synergies and combinatorial approaches with available immunotherapy agents in the clinical management of metastatic disease.

## 6 A Proximal Oncology: Pioneering a proximal and accessible locus of cell cycle control

**FIGURE 3:**
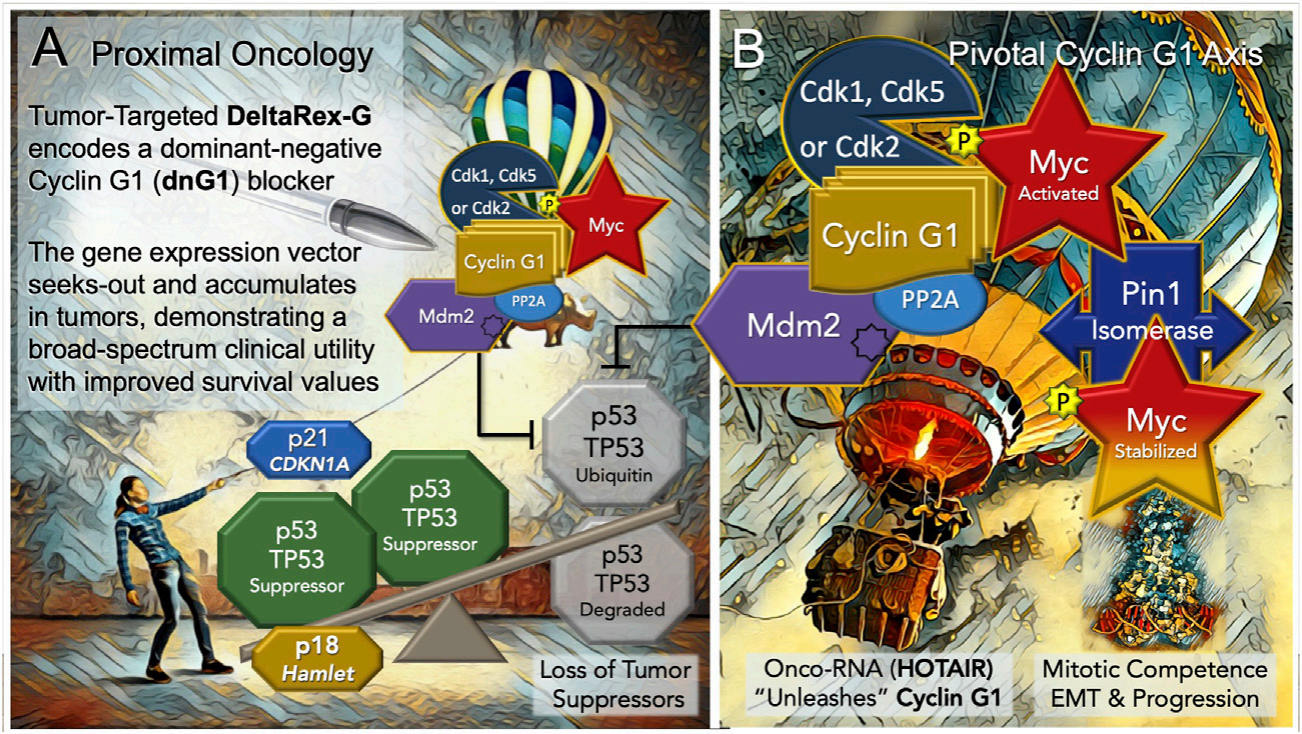
Blocking the “Cyclin G1 Axis” of Interacting Oncogenes and Tumor Suppressors. **(A)** DeltaRex-G, encoding a cytocidal ‘dominant negative’, i.e. a truncated construct (dnG1) of the executive cyclin G1 oncogene (CCNG1), is shown as a molecular-genetic “silver bullet,” blocking the Pivotal Cyclin G1 Axis of associated oncogenes, which is activated by signal transduction and/or loss of natural tumor suppressors (p53 (TP53); p21, CDKN1A; and p18, Hamlet). **(B)** The Cyclin G1 Axis of interacting oncoproteins include Cyclin G1, Myc, Cdk2, Cdk5 or Cdk1, Mdm2, and the proline-directed/phosphorylation-dependent Pin1 isomerase, which stabilizes the Cyclin G1/Cdk-activated Myc oncoprotein by phosphorylation (at serine 62) and maintains its transcriptional bioactivity. Note: loss of the natural tumor suppressors—by 1) mutations in p53, 2) loss of regulatory miRNAs, 3) by viral subversion (e.g., miR-122), or 4) by a growing number of oncogenic-RNAs (e.g., HOTAIR)— “unleashes” the expression and functions of the Cyclin G1 Axis which lead to stem cell activation, tumorigenesis, error-prone DNA replication, increasing oncogene addictions, EMT/metastasis, and progression of cancers.

Discerning the upstream signaling pathways that link cell growth and DNA replication to cell cycle progression can be a daunting endeavor in a landscape where the vast numbers of cell cycle checkpoint regulators and their myriad of protein-protein interactions (PPIs) reach a zillion (Yasutis and Kozminski, 2013). It is in this regard, that DeltaRex-G stands alone in defining a single proximal (gene locus) and a strategic point of cell cycle control (dnG1 blockade) suitable for broad-spectrum cancer gene therapy. Thus, the Pivotal Cyclin G1 axis was conferred as a uniquely useful and accessible point of clinical cell cycle control, with a review of molecular mechanisms for oncologists (Al-Shihabi et al., 2018; Gordon et al., 2018)—demonstrating long-term survival benefits of a Cyclin G1 pathway blocker in a broad spectrum of advanced cancers, when provided as monotherapy or combined with adjunctive immunotherapy; empowering modern medical oncologists to address persistent problems of chemotherapy resistance, recurrence, and occult progression of metastatic disease. An updated diagram of the key oncogenic drivers of the Pivotal Cyclin G1 Axis is shown in Figure 3, presenting the Cyclin G1/Cdk/Myc/Pin1/Mdm2 as a fundamental set of interacting proto-oncogenes—which acting together create a stabilizing/reinforcing molecular-genetic *flywheel,* exhibiting momentum, in terms of oncoprotein stability—thus, maintaining stem cell *competence* and stem *cell survival* programs: from the beginnings of stem cell activation from quiescence (Go/G1), through the phases of the cell division cycle, and further, with mechanistic links extending from the checkpoint guardians of DNA fidelity to cellular differentiation and fate. In normal development, p53 tumor suppressor and Cyclin G1cooperate in mediating genome stability in somatic cells, both being required for DNA repair, with Cyclin G1 playing a positive role in the coordination of cell growth and cell proliferation (Faradji et al., 2011; Bayer et al., 2017). Follow-up studies of archived tumor samples in a long-term survivor (>12 years) of metastatic pancreatic cancer following repeated infusions of DeltaRex-G, revealed a p53 (TP53) mutation (*G199V*) associated with an increased anti-apoptotic survival function, affirming the therapeutic efficacy of DeltaRex-G in the background of p53 suppressor mutations (Morse et al., 2021). Indeed, recent studies have confirmed that the Cyclin G1 (CCNG1 proto-oncogene) is upregulated by mutations in tumor protein p53 (TP53), which is associated with both tumorigenesis and tumor progression (Xu et al., 2019).

The Cyclin G1 proto-oncogene is known to be activated and up-regulated by mutation and viral subversion, as well as a growing number of long-noncoding oncogenic RNAs, and mitogenic signal transduction. Notably, the hepatitis B virus HBx protein deregulates cell cycle checkpoint controls and enhances hepatoblastoma cell proliferation, in part, by suppressing miR-122, a predominant negative regulator of Cyclin G1 (CCNG1) expression, which is lost in the process of neoplastic transformation and oncogenesis (Bandopadhyay et al., 2016). Recent, studies of experimental Sendai virus infection in murine embryo fibroblasts revealed new molecular mechanisms by which NF-kB signaling components (p50 and p65 subunits) are recruited to the cell nucleus (see Figure 2) to directly activate and up-regulate both Cyclin G1 (CCNG1) and p21 (CDKN1) at the level of gene expression, enhancing the transcription of both genes upon virus infection (Burns and Kerppola, 2022). A sharp focus at the level of the human Cyclin G1/CCNG1 promoter reveals a DNA regulatory cis-element (tandem TCTCGCGAGA motif) that is, limited to approximately 5% of human genes, particularly those involved in enhanced protein synthesis and transition phases of the cell division cycle (Wyrwicz et al., 2007). Remarkably, the Dyrk1a protein kinase was revealed as a gene-specific RNA Polymerase II kinase (Di Vona et al., 2015), which selectively recognizes the aforementioned palindromic DNA motif, which is also clearly present at the level of the human CCNG1 promoter.

The CCNG1 gene itself is subject to dysregulation by the action of oncogenic long-noncoding RNAs (Cheng et al., 2018; Li et al., 2019; Abula et al., 2022); and by direct/activating mutations. A close examination of the pathogenesis of Burkitt’s lymphoma in a screening for IG-translocations to non-MYC partners that could cooperate with MYC in pathogenesis, identified a telltale IGK-GGNG1 translocation: a chromosomal breakpoint at the CCNG1 promoter that had apparently occurred earlier (at pre-B or immature B Cell stage) in the evolution of the tumor, which suggested that IG-MYC translocations may not always be the initial genetic event in Burkitt’s lymphoma (López et al., 2019). Focused studies of IGH rearrangements in myeloid neoplasms identified an IGH-CCNG1 breakpoint translocation at the cyclin G1 (CCNG1) promoter, which demonstrated overexpression of CCNG1 in the patient’s bone marrow (Xiao et al., 2020). The patient was diagnosed with chemotherapy-related acute myeloid leukemia and succumbed to the disease 2 months later. The authors of the study identified Delta-Rex-G as a potential intervention, noting its impressive results regarding long-term cancer-free survival in clinical trials, and suggesting clinical evaluation of the anti-CCNG1 strategy as a treatment in such cases with very poor prognosis.

One can now extend the epic of gene discovery from Hera to Hamlet: that is, from the p38 (MAPK14), kinase that is, at times, physically associated with the EGF-receptor to p18 Hamlet, a highly conserved protein ‘*sensor,*’ partner of both p53 and Cyclin G1, and critical governor of cell fate. The “Hamlet” gene, originally cloned and characterized by Xu et al. as a novel Cyclin-G1- binding protein: exhibits both a nuclear targeting motif and a zinc-finger motif commonly found in transcription factors (Xu et al., 2000). It turns out that p18 FX3/Hamlet (originally FX3, aka ZNHIT1, aka Hamlet) is directly phosphorylated and regulated by the p38 stress-related proline-directed Hera kinase/MAPK14 (Cuadrado et al., 2007). Indeed, p18 Hamlet interacts with p53, as well as Cyclin G1, and activates p53-dependent gene promoters. Normally tightly regulated by Cyclin G1, which induces its degradation, p18 Hamlet accumulates in response to genotoxic stresses such as UV or cisplatin treatment, and thereby acts as a sensor which mediates p53-dependent responses to different genotoxic stresses (Lafarga et al., 2007)—balancing the molecular mechanisms between cell cycle arrest (and DNA 216 repair) by the p53-dependent up-regulation of p21^Cip1^ (CDKN1), or the equally dramatic alternative: death by apoptosis, *via* p53-dependent induction of proapoptotic promoters. In terms of the Proximal Oncology presented herein (Figure 3), p18 Hamlet physically links p38 Hera/MAPK14 to the complex regulation and functions of the p53 tumor suppressor (Vousden and Prives, 2009), thereby defining a prospective tumor suppressor that is, essentially lost, as is p53, with oncogenic activation and overexpression of the Pivotal Cyclin G1 (CCNG1) Axis.

Of considerable relevance and conceptual support for this highly simplified model of the Cyclin G1 Axis are comparative screens identifying genetic variants in cell cycle and checkpoint control pathways that confer particular susceptibility to aggressive cancers: Notably, CCNG1, CDK2, CDK5, and MDM2 were identified among the Top-7 genes with variants associated with aggressive prostate cancer (Kibel et al., 2016). Likewise, comparatively high expression of the mitogen-activated protein kinase kinase 3, MKK3 (see Figure 2), which activates Myc transcription, is associated with an increased incidence of triple-negative breast cancer and a worse clinical outcome (Yang et al., 2020). Armed with the enabling potentiality of tumor-targeted gene delivery, with a broad spectrum bioactive, in the embodiment of DeltaRex-G, the challenge remains to match the precision medicine with the patient’s tumor burden in a timely manner (Ravicz et al., 2021), with thoughts of cancer control and improved long-term outcome in mind. In closing, we reflect upon the development of new Clovis Point platforms for the delivery of future medicines, including the tumor-targeted delivery small molecules (e.g., paclitaxel), monoclonal antibodies, and future RNA-based therapeutics. Taken together, the concepts of collagen binding growth factors, autologous stem cell capture, and pathotropic targeting have provided new insights and molecular-genetic tools for wound healing, tissue regeneration, and cancer control.
